# Dépôts cornéens de ciprofloxacine en post chirurgie de cataracte

**DOI:** 10.11604/pamj.2018.31.135.16232

**Published:** 2018-10-23

**Authors:** Ghita Bouayad, Abdelbarre Oubaaz

**Affiliations:** 1 Université Mohammed V Souissi, Service d’Ophtalmologie de l’Hôpital Militaire d’Instruction Mohamed V, Rabat, Maroc

**Keywords:** Dépôts, cornée, ciprofloxacine, Deposits, cornea, ciprofloxacin

## Image en médecine

We here report the case of a 62-year old patient who had undergone left eye phacoemulsification cataract surgery with good outcome. In the postoperative period he had received treatment with ciprofloxacin ophthalmic (eye drops). The patient had been lost to follow-up for 2 months. He hadn’t stopped therapy during these months. He presented with paracentral white crystalline opacity, corresponding to corneal deposit of ciprofloxacin. The therapy with antibiotic eye drops was immediately suspended. The patient underwent very superficial surgical debridement and received wetting agents at high doses with complete ulcer healing at the level of the corneal deposit. However he continued to have epithelial inferior parapupillary opacity without visual impairment.

Nous rapportons le cas d’un patient âgé de 62 ans, opéré de cataracte de l’œil gauche par phacoémulsification avec suites simples; il a reçu en post opératoire un traitement comprenant la ciprofloxacine en collyres. Le patient a été perdu de vue pendant 2 mois sans arrêter le traitement; il se présente avec une opacité para-centrale, blanche, d’allure cristalline, correspondant à un dépôt cornéen de ciprofloxacine. Le collyre antibiotique a été immédiatement suspendu et le patient a bénéficié d’un débridement chirurgical très superficiel, et a été mis sous agents mouillants à fortes doses, avec cicatrisation complète de l’ulcération en regard du dépôt cornéen, mais il a gardé une opacité épithéliale para-pupillaire inférieure ne gênant toutefois pas la vision.

**Figure 1:**
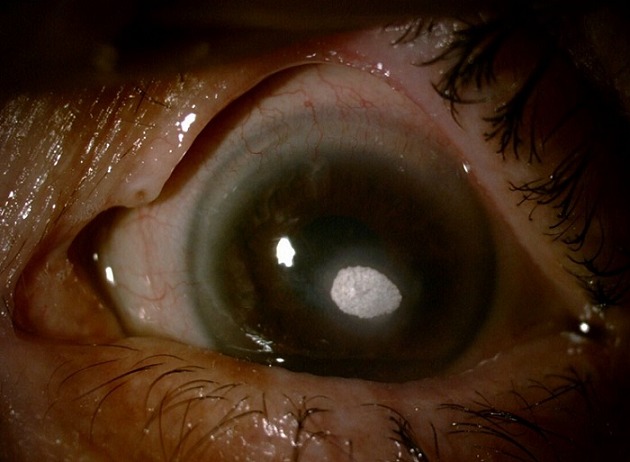
dépôt cornéen de ciprofloxacine

